# Inbreeding patterns and genetic diversity under selection in Teha sheep

**DOI:** 10.3389/fgene.2025.1576125

**Published:** 2025-06-27

**Authors:** Shunzhe Wang, Long Liang, Dilinigeer Ziyayiding, Wenjing Jiao, Hailati Kasimu, Sangang He, Mingjun Liu

**Affiliations:** ^1^ Key Laboratory of Animal Biotechnology of Xinjiang, Key Laboratory of Genetics Breeding and Reproduction of Grass Feeding Livestock, Ministry of Agriculture and Rural Affairs of the People’s Republic of China, Institute of Biotechnology, Xinjiang Academy of Animal Science, Urumqi, China; ^2^ Institute of Animal Husbandry and Veterinary, Zhejiang Academy of Agricultural Sciences, Hangzhou, China

**Keywords:** genetic diversity, genomic inbreeding coefficient, ROH, Teha sheep, candidate genes

## Abstract

**Background:**

Inbreeding and genetic diversity are critical factors influencing the adaptability, productivity, and sustainability of livestock populations. Teha sheep, a crossbred line between Texel and Kazakh sheep, are an important meat-producing breed in China, yet their genetic structure and inbreeding status remain underexplored. In this study, we aim to evaluate inbreeding coefficients, genetic diversity, and selection signatures in Teha sheep by integrating pedigree and genomic data.

**Results:**

Analysis of pedigree data from 2,652 individuals revealed a low inbreeding coefficient (FPED = 0.001), whereas analysis of genomic data from 1,271 individuals indicated slightly higher inbreeding coefficients, with the FROH averaging 0.044. Genetic diversity metrics, including Ho = 0.347 and PIC = 0.345, confirmed moderate variability within the population. A significant region of runs of homozygosity (ROH) hotspot was identified on chromosome 2 (112.01–119.89 Mb), encompassing genes such as *MSTN*, *TUBGCP5*, and *NIPA2*, which are associated with muscle growth, fat metabolism, and skeletal development. Notably, *CYFIP1*, *SAP130*, and *UGGT1* were identified as key genes shared across ROH hotspots, QTL regions, and LD blocks, implicating their roles in growth efficiency, carcass quality, and protein regulation under stress. These findings reveal critical genomic regions contributing to the breed’s productivity and adaptability.

**Conclusion:**

In this study, we highlight the low inbreeding levels and moderate genetic diversity of Teha sheep, emphasizing the integration of pedigree and genomic analyses for sustainable breeding programs. The identification of key genes provides a foundation for optimizing productivity and maintaining genetic variability in this important livestock population.

## Introduction

Inbreeding refers to the probability that two alleles at a given locus are identical by descent, resulting from the mating of related individuals. In livestock populations, inbreeding commonly arises due to directional selection, small effective population sizes, or closed breeding systems (Falconer, 1996; Keller et al., 2011). Elevated levels of inbreeding increase genome-wide homozygosity, which may lead to the expression of deleterious recessive alleles and a decline in overall fitness, a phenomenon known as inbreeding depression (Doekes et al., 2020). This condition is typically characterized by reduced fertility, impaired immune function, and decreased production performance in farm animals (Doekes et al., 2020; Forneris et al., 2021). Accurate estimation of inbreeding is therefore essential for controlling genetic risks in breeding programs. Traditionally, inbreeding has been quantified using FPED, which estimates the expected proportion of autozygous loci based on known genealogical relationships (Falconer, 1996). However, the accuracy of FPED is highly dependent on pedigree completeness and depth, and it often fails to reflect actual genomic inbreeding, especially in populations lacking systematic pedigree recording (Howard et al., 2017).

Advancements in genomic technologies have revolutionized the assessment of inbreeding and population structure in livestock. High-throughput SNP genotyping platforms have enabled the precise quantification of genomic inbreeding coefficients by leveraging dense marker coverage to detect homozygous segments across the genome. These detected homozygous segments, particularly runs of homozygosity (ROH), represent regions likely to be IBD, allowing research workers to infer autozygosity (Chang et al., 2015). Unlike pedigree-based approaches, which estimate the probability of IBD based on known relationships and can be limited by pedigree completeness and accuracy, genomic methods directly detect patterns of genetic variation (such as ROH) to assess realized homozygosity arising from both recent and ancient common ancestors, offering robust insights into inbreeding dynamics. These approaches also facilitate the detection of genomic regions under selection and elucidate breed-specific evolutionary trajectories (Xiang et al., 2016). In sheep, high-throughput genotyping and ROH analysis have been instrumental in characterizing genetic diversity and identifying signatures of recent and ancestral inbreeding, informing sustainable breeding practices (He et al., 2020). Additionally, ROH-based metrics provide nuanced perspectives on the genomic distribution of inbreeding, distinguishing short ROH (indicative of ancient inbreeding) from long ROH (reflecting recent inbreeding), thus enhancing the precision of genetic management strategies (Van Der Valk et al., 2019).

In recent years, studies on crossbred populations such as Red Angus × Chinese Red Steppe cattle and Yunnan semi-fine wool sheep have begun to explore their genetic structure using genomic data (Guo et al., 2022; Rostamzadeh Mahdabi et al., 2025). These studies demonstrate that ROH analysis is not only effective for evaluating inbreeding levels but also capable of revealing historical recombination patterns and potential selection regions. This provides a foundation for understanding genetic diversity in crossbred populations and offers insights for genomic evaluation approaches in novel hybrid breeds.

Teha sheep, a crossbred population derived from Texel rams and Kazakh ewes, are predominantly raised in the Yili region of Xinjiang, China, and valued as a meat-producing breed with strong local adaptation. Combining the rapid growth rate and high meat productivity of the Texel breed with the superior resilience and adaptability of Kazakh sheep, Teha sheep have become a key resource for lamb production in the regional sheep industry. However, intensive selection and limited population size may elevate inbreeding levels, potentially leading to inbreeding depression that affects the growth rate and reproductive traits (Falconer, 1996).

The primary objective of this study was to evaluate the genetic diversity and inbreeding levels of the Teha sheep population using pedigree and genomic data. The findings derived from this research are intended to provide valuable insights for guiding the sustainable breeding and genetic management strategies of this breed.

## Materials and methods

### Study population

Teha sheep, a meat-producing crossbred derived from Texel rams and Kazakh ewes, were sourced from the Sheep Nucleus Stud Farm in Zhaosu County, Yili, Xinjiang, China. The current population has been under selection since 2018, primarily for growth performance and meat yield. A total of 2,652 individuals were analyzed, that is, 321 rams and 2,331 ewes, with complete pedigree records spanning five generations (2018–2023). The number of ewes was evenly distributed across the years, whereas most rams were born between 2022 and 2023. This sampling ensured adequate representation across reproductive years, particularly for maternal lines, enabling reliable estimation of relatedness and inbreeding levels.

### Sample collection and data

Genomic analysis involved 1,273 Teha sheep, randomly sampled from the 2,652 pedigree population, retaining potential relatedness to support inbreeding and genetic diversity studies. Blood samples were collected from each sheep, with DNA extracted using a commercial kit. Genomic genotyping was performed using the Illumina OvineSNP50 BeadChip. To enhance the analysis of economic traits, the chip was supplemented with custom single-nucleotide polymorphism (SNP) markers relevant to economic traits, yielding 64,734 markers. Genotyping was conducted by Beijing Compass Biotechnology Co., Ltd.

### SNP genotyping and quality control

Genomic data were obtained from 1,273 individuals genotyped with 64,734 SNP markers distributed across 26 autosomes. Quality control was conducted using PLINK v1.90 software, with the following criteria applied (Chang et al., 2015): (1) SNPs with a missing call rate >5% were excluded, (2) SNPs with minor allele frequency (MAF) < 0.01 were removed, (3) SNPs deviating from the Hardy–Weinberg equilibrium (HWE) (p < 1 × 10^−6^) were excluded, and (4) samples with a genotyping success rate < 90% were removed. After quality control, 1,271 samples and 56,297 high-quality SNPs were retained for subsequent analyses.

### Genetic diversity evaluation

The genetic diversity of the Teha sheep population was assessed using six parameters derived from SNP data: Ne, Ho, He, PIC, MAF, and ENA.

Ne, chosen for its ability to track historical population dynamics sensitive to inbreeding, reflects the risk of inbreeding and genetic drift, and it was estimated *via* LD using the formula (Waples and Do, 2010):

$$\text{Ne}=\left(1/4c\right)\times \left(1/r^{2}-1\right),$$

where *r* ^2^ is the LD value between SNP loci and *c* is the genetic distance between loci measured in centimorgans (cM). Default parameters of PLINK were used for calculations.

Ho and He were selected to assess heterozygosity loss due to inbreeding by comparing actual and potential genetic variation (Nei, 1973):

$$\text{Ho}=\frac{1}{N}\sum_{k=1}^{N}\frac{H_{k}}{n},\text{He}=\frac{2_{n}}{2n-1}\frac{1}{N}\sum_{k=1}^{N}1-\sum p_{\text{ki}}^{2},$$

where N is the total number of loci, *H*_*k*_ is the number of heterozygous individuals at locus *k*, *n* is the population size, and *p*_*ki*_ is the frequency of allele *i* at locus *k*.

PIC, chosen for its utility in evaluating the informativeness of genetic markers in inbreeding, indicates reduced marker diversity due to inbreeding-driven fixation, and it was calculated as follows (Botstein et al., 1980):

$$\text{PIC}=1-\sum_{i-1}^{n}P_{i}^{2}-\sum_{i=1}^{n-1}\sum_{j=i+1}^{n}2P_{i}^{2}P_{j}^{2},$$

where *p*_*i*_ and *p*_*j*_ are the allele frequencies at each locus and *n* is the number of alleles.

MAF, chosen to ensure reliable SNP data for inbreeding analysis by filtering low-frequency alleles, supports accurate detection of inbreeding-related diversity loss, and it was computed with a threshold of >0.01 (Chang et al., 2015).

ENA was calculated using PLINK v1.90. This parameter reflects the number of alleles required to achieve the observed level of heterozygosity in a population, assuming equal allele frequencies.

### Detection and classification of ROH

ROH in the sample were identified using PLINK v1.90 software. The criteria for ROH identification included the following: (1) a minimum ROH length of 1 Mb; (2) at least 50 SNPs within an ROH, ensuring result reliability and reducing false positives; (3) allowance for one SNP per 100 kb, aligning with the chip’s average SNP spacing; and (4) a maximum of one missing genotype allowed per sliding window, tolerating minor genotyping errors (e.g., copy number variations) and enhancing detection stability (Meyermans et al., 2020; Arias et al., 2022). All ROH segments were categorized into four classes based on length: 1–5 Mb, 5–10 Mb, 10–20 Mb, and >20 Mb (Ferencakovic et al., 2011; Paim et al., 2020; Zayas et al., 2024).

### Assessment of genomic inbreeding coefficients

FPED values were estimated using ASReml-R software based on pedigree information. The relationship matrix (*A*) was constructed from the pedigree data, and its inverse was utilized to calculate the coefficients. The calculation followed the formula FPED = diag(*A*)- 1, where *A* represents the relationship matrix (Butler et al., 2017).

SNP-based genomic inbreeding coefficients (FHAT1, FHAT2, FHAT3, and FHOM) were calculated using PLINK software, reflecting different aspects of genomic inbreeding (Chang et al., 2015; Dadousis et al., 2022). Specifically, FHAT1 estimates inbreeding as the excess variance standardization, whereas FHAT2 quantifies excess homozygosity, which is analogous to Fhet. The FHAT3 coefficient measures the correlation between alleles within gametes. FHOM represents the proportion of observed homozygous genotypes relative to the expected number under the Hardy–Weinberg equilibrium.

The FROH was determined to evaluate inbreeding patterns derived from extended homozygous regions in the genome. The FROH was calculated as FROH = ∑ LRon/LAuT, where ∑ LRon represents the total length of ROH identified in an individual’s genome and LAuT refers to the total length of the autosomal genome. Furthermore, chromosome-specific inbreeding coefficients (FROHOAR) were calculated as FROHOAR = LROHOAR/LoAR, where LROHOAR denotes the ROH length within a specific chromosome and LoAR is the total autosomal length for that chromosome.

### Identification of ROH hotspots and functional annotation

To identify potential ROH hotspot regions, the percentage of SNP occurrence within ROHs was calculated by dividing the number of animals in which a specific SNP occurred by the total number of animals analyzed. The SNP positions were plotted across the genome, and genomic regions with the top 1% SNP occurrence were considered as potential ROH hotspots (Ferencakovic et al., 2013).

Annotated genes located within the identified ROH hotspots were identified and annotated based on the *Ovis aries* reference genome (Oar_v3.1) using GFF annotation files obtained from the National Center for Biotechnology Information (NCBI).

### LD block and QTL annotation analysis

The LD structure within the ROH hotspot regions was analyzed using LDBlockShow v1.40 (Dong et al., 2021). LD heatmaps were generated based on SNP data from chromosome 2 (112,011,937–119,890,211 bp) to visualize the patterns of linkage disequilibrium and identify significant LD blocks within the hotspot regions.

To investigate potential associations between the ROH hotspot regions and economically important traits, we first defined the genomic coordinates of the LD regions, QTL regions, and ROH hotspot regions. QTL annotations were retrieved from the Sheep QTL Database (SheepQTLdb) (https://www.animalgenome.org/cgi-bin/QTLdb/OA/index). This database provides curated QTL data based on the *O. aries* genome assembly OAR3.1. Genomic coordinates of the hotspot regions were cross-referenced with the QTL data to identify relevant QTLs.

Subsequently, to identify genes within these defined regions (including LD regions, QTL regions, and ROH hotspot regions), we performed extraction using bcftools v1.12 (https://samtools.github.io/bcftools/bcftools.html) in conjunction with the genome annotation file (GFF format) for the OAR3.1 genome. This integration facilitated the annotation of traits associated with ROH hotspot regions and further understanding of their potential biological background through the identified genes.

## Results

### Genetic diversity

**FIGURE 1:**
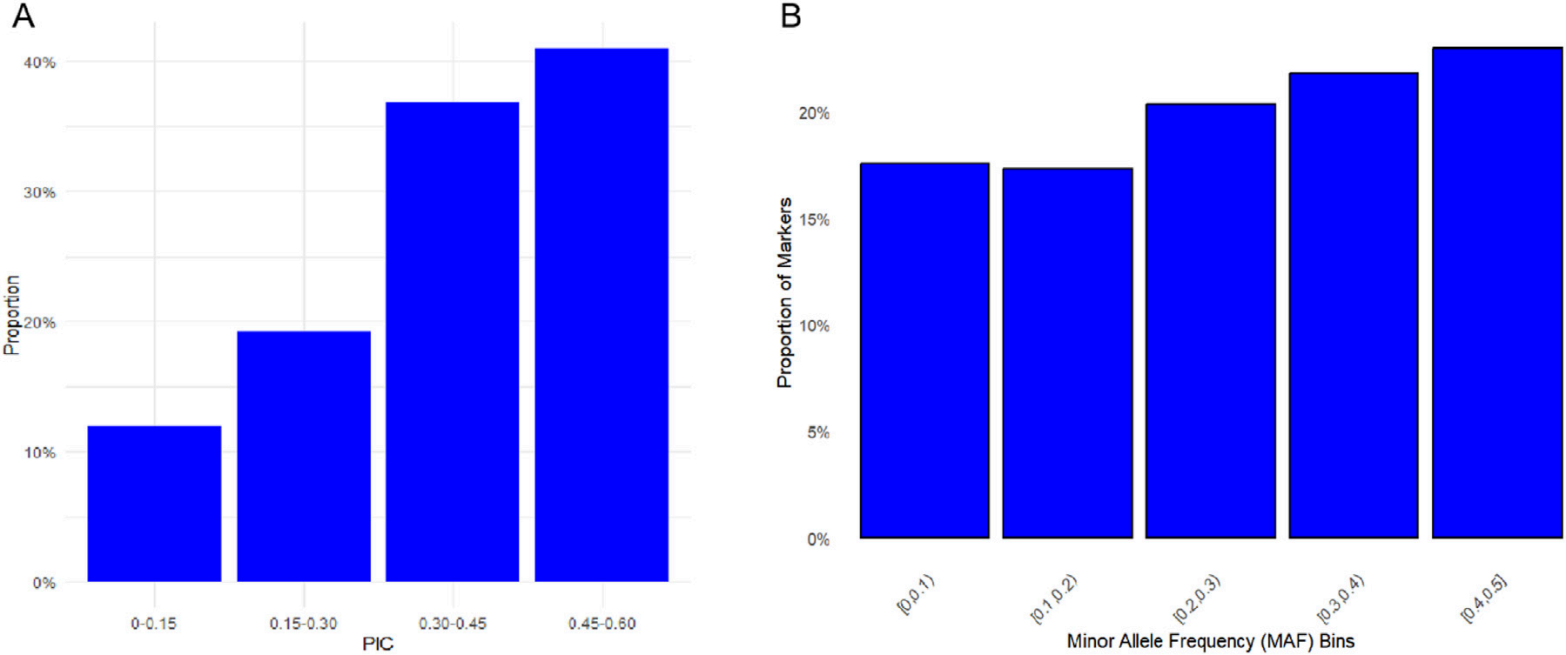
Distributions of genetic diversity parameters among SNPs in the Teha sheep. **(A)** Distribution of polymorphic information content (PIC) values across SNP markers. PIC values were grouped into defined intervals to reflect marker informativeness. **(B)** Minor allele frequency (MAF) distribution showing the proportion of SNPs in each frequency bin.

Six diversity parameters were calculated based on SNP data (Table 1). The effective population size (Ne) was 214.893. Both the observed heterozygosity (Ho) and expected heterozygosity (He) were 0.347. The mean polymorphic information content (PIC) was 0.345. The effective number of alleles was 1.602. The minor allele frequency (MAF) was 0.264. The PIC distribution showed 40% of SNPs with values 0.45–0.60, 35% with 0.30–0.45, 20% with 0.15–0.30, and 10% with <0.15 (Figure 1A). The MAF distribution is presented in Figure 1B.

**TABLE 1:** Genetic diversity parameters calculated in a population of Teha sheep.

Index	Average value
Ne	214.893
PIC	0.345
He	0.347
Ho	0.347
ENA	1.602
MAF	0.264

Abbreviations: Ne, effective population size; PIC, polymorphic information content; He, expected heterozygosity; Ho, observed heterozygosity; ENA, effective number of alleles; MAF, minor allele frequency.

## Analysis of ROH

**FIGURE 2:**
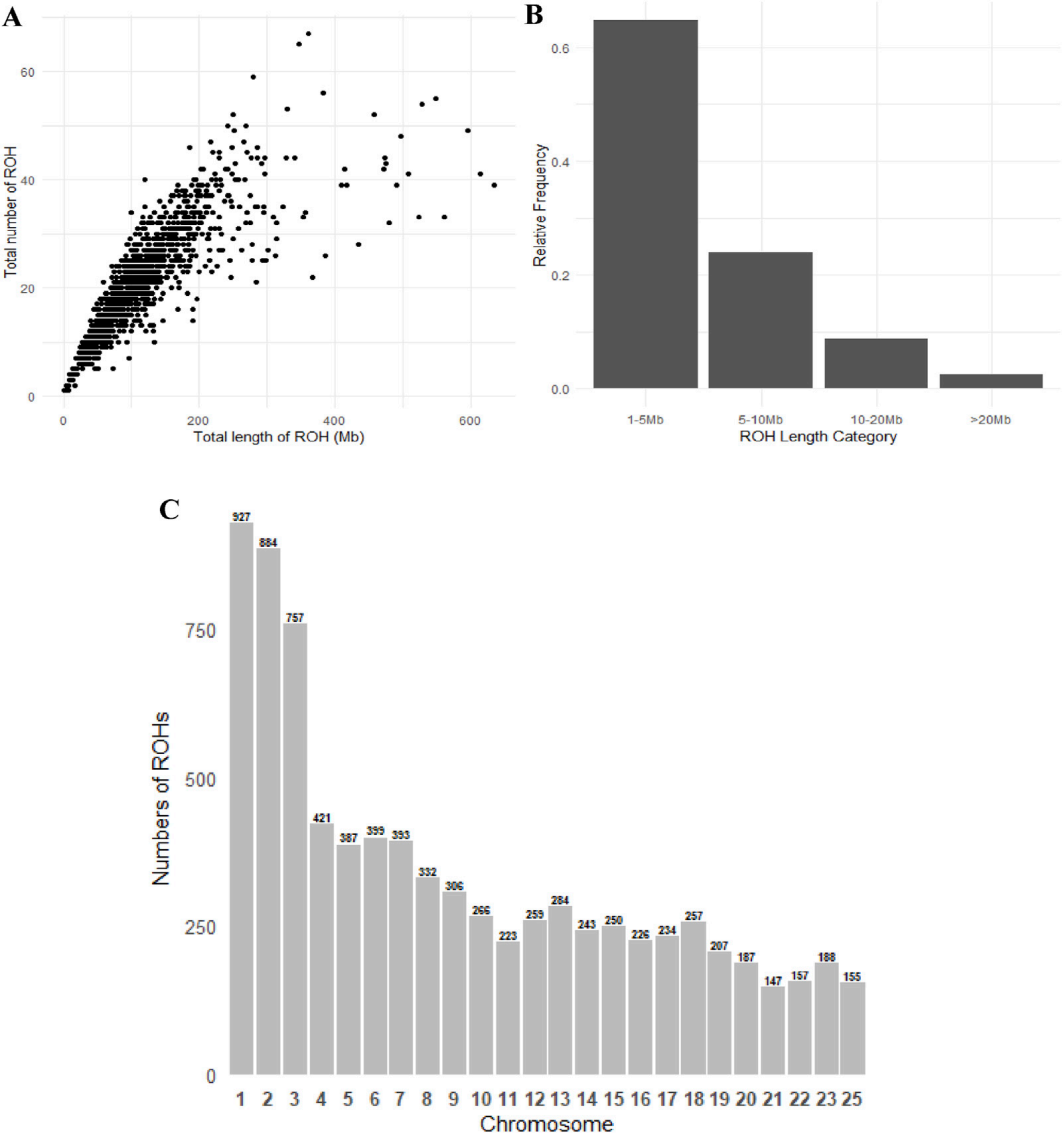
Patterns of runs of homozygosity (ROH): **(A)** scatterplot showing the correlation between the number of ROH segments and the total ROH length per individual, **(B)** proportion of ROH segments falling within different length categories, and **(C)** distribution of ROH in chromosomes.

A total of 26,560 ROH segments were identified across 1,271 samples from the Teha sheep population. These segments exhibited individual ROH lengths ranging from 1.01 Mb to 103 Mb. The average ROH length was 3.06 Mb. The number of ROH segments was correlated with the total length covered by ROHs (Figure 2A).

ROH segments were distributed as follows: 1–5 Mb (35.6%), 5–10 Mb (29.7%), 10–20 Mb (21.1%), and >20 Mb (13.6%) (Table 2). Variation in the number of ROH segments was observed across chromosomes. Chromosome 1 exhibited the highest number of ROH segments (3,549), whereas chromosome 25 exhibited the lowest (451). Other chromosomes displayed intermediate counts, reflecting differences in chromosomal architecture (Figure 2C).

**TABLE 2:** Distribution of runs of homozygosity (ROH) of different lengths in Teha sheep.

Category	ROH (N)	ROH (%)	Max	Min	Mean	SD
ROH 1–5 Mb	17209	35.6	5	1.01	3.06	0.9
ROH 5–10 Mb	6370	29.7	10	5	6.9	1.4
ROH 10–20 Mb	2313	21.1	20	10	13.5	2.7
>20 Mb	668	13.6	103	20	30.2	11.1

Abbreviations: ROH (N), number of ROH; ROH (%), percentage of ROH; Min, minimum; Max, maximum; SD, standard deviation.

Recognizing the potential influence of data filtering, we conducted a complementary ROH analysis wherein MAF and HWE filters were not applied. The outcomes of this parallel investigation largely mirrored the patterns and significant findings observed in the filtered dataset. Our decision to employ MAF and HWE filters in the final analysis was grounded in their established role within genomic studies for data quality control, specifically in eliminating low-quality or potentially erroneous SNPs, thereby ensuring the accuracy and broader applicability of our findings.

### Pedigree and genomic inbreeding analysis

The basic statistics for inbreeding coefficients based on pedigree and genomic data are presented in Table 3. The pedigree-based inbreeding coefficient (FPED) ranged from 0 to 0.25, with an average of 0.001, indicating a relatively low level of inbreeding within the Teha sheep population. The inbreeding coefficient derived from FROH ranged from 0.0004 to 0.241, with a mean value of 0.044 and a standard deviation of 0.031, demonstrating that most individuals exhibited low FROH values. However, a small subset exhibited higher FROH values, approaching 0.18. The SNP-based inbreeding coefficients (FHAT1, FHAT2, FHAT3, and FHOM) exhibited distinct distributions, with average values close to zero. Among these, FHAT1 displayed the widest range, which was from −0.228 to 0.342 (Table 3).

**TABLE 3:** Statistics of inbreeding coefficients based on pedigree and genomic information.

Method	Min	Max	Mean	Median	SD	P1	P99
FPED	0	0.25	0.001	0	0.013	0	0
FROH	0.0004	0.241	0.044	0.038	0.031	0.00087	0.180
FHAT1	−0.228	0.342	−0.050	−0.052	0.064	−0.166	0.120
FHAT2	−0.206	0.268	−0.006	−0.008	0.061	−0.144	0.177
FHAT3	−0.123	0.212	−0.006	−0.009	0.028	−0.064	0.101
FHOM	−0.165	0.231	−0.007	−0.012	0.041	−0.083	0.150

Abbreviations: Min, minimum; Max, maximum; SD, standard deviation; P1, first percentile; P99, 99th percentile.

**FIGURE 3:**
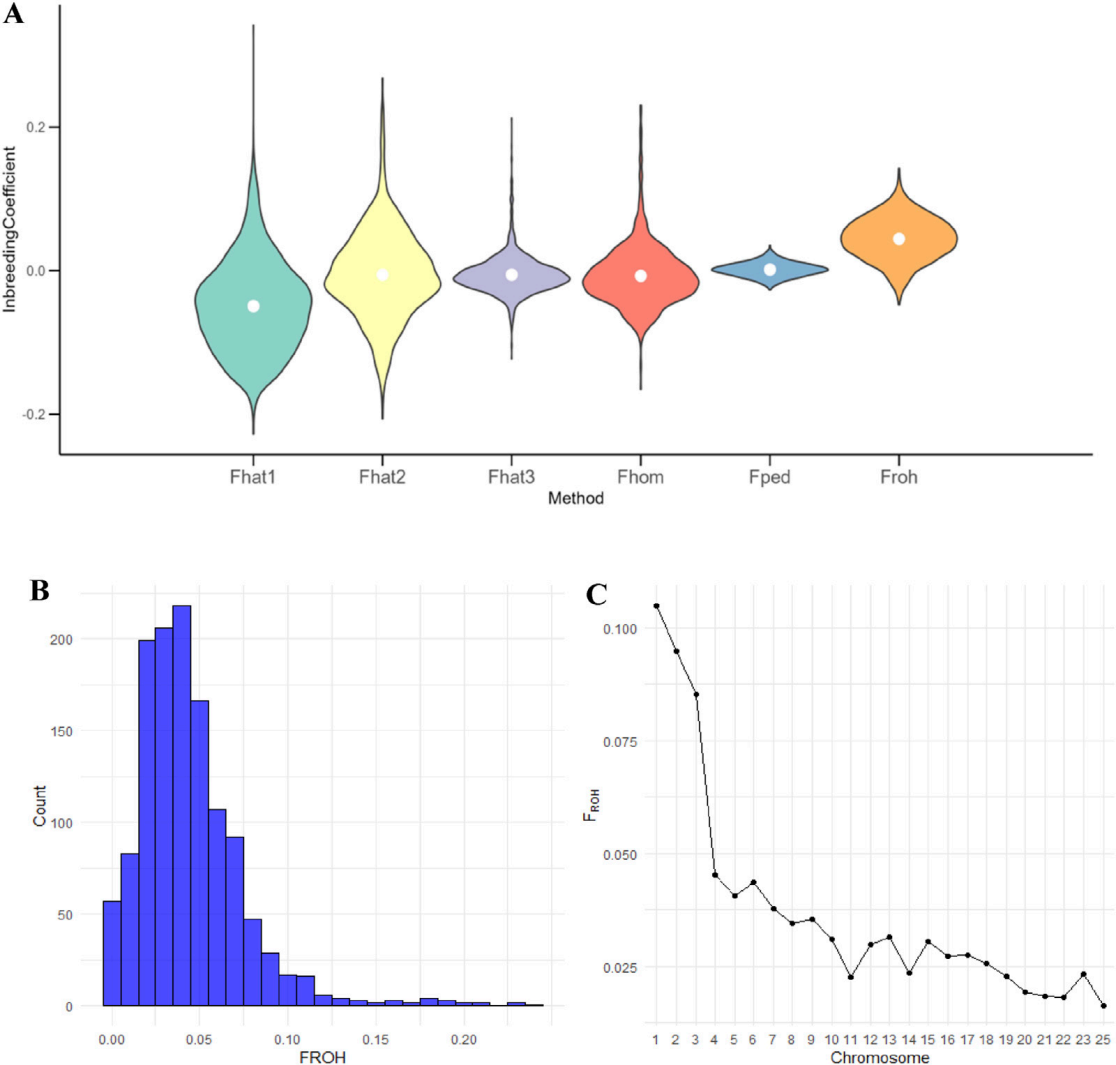
Distribution and patterns of inbreeding coefficients of Teha sheep: **(A)** violin plot illustrating the distribution of inbreeding coefficients (inbreeding coefficient based on excess variance standardization (FHAT1), inbreeding coefficient based on excess homozygosity (FHAT2), inbreeding coefficient based on allele correlation within gametes (FHAT3), inbreeding coefficient based on the proportion of homozygous genotypes (FHOM), pedigree-based inbreeding coefficient (FPED), and runs of homozygosity-based inbreeding coefficient (FROH)) calculated using different methods. The width of each violin represents the density of the coefficient values and the white dot indicates the mean. **(B)** Histogram showing the distribution of FROH values across all individuals. **(C)** Chromosomal distribution of FROH values, where each point represents the mean FROH in a chromosome.

The violin plot showed the distribution of inbreeding coefficients calculated using different methods. FHAT1 had the largest distribution range, whereas FHOM had the smallest range, and FPED exhibited a concentrated distribution (Figure 3A).

The histogram of FROH values revealed that the majority of individuals fell within the 0–0.05 range, with only a few reaching 0.2, underscoring the overall low level of inbreeding within the Teha sheep population (Figure 3B). Chromosomal-level analysis showed variability in FROH across chromosomes, with chromosome 25 exhibiting the lowest FROH value, averaging only 0.01 (Figure 3C).

### Genomic regions with ROH hotspot

**FIGURE 4:**
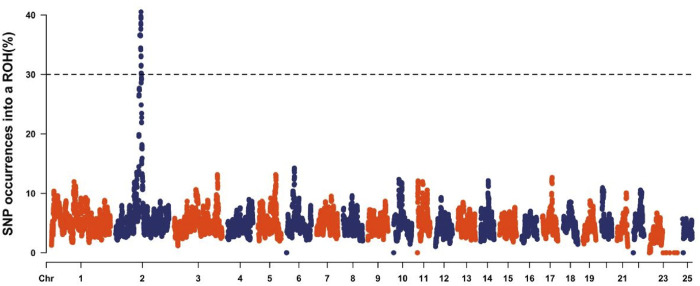
Manhattan plot of the occurrence of single-nucleotide polymorphisms (SNPs) in runs of homozygosity (ROH) islands across different chromosomes. The horizontal line corresponds to the threshold calculated as SNPs with a p-value of ROH incidence higher than 0.999.

Analysis of the Teha sheep genome identified a prominent ROH hotspot on chromosome 2, spanning positions 112,011,937 to 119,890,211 bp. This region covered 7.88 Mb and contained 184 SNPs (Figure 4; Table 4).

**TABLE 4:** Candidate regions and genes identified from the ROH hotspot in Teha sheep.

Chr	Start bp	End bp	nSNPs	Length (Mb)	Relevant genes
2	112011937	119890211	184	7.88	AMER3, AMMECR1L, and ANKAR ARHGEF4, ASNSD1, BIN1, C2H2, CCDC115, COL3A1, and COL5A2 CYFIP1, ERCC3, and FAM168B GULP1, HIBCH, HS6ST1, and IMP4 INPP1, IWS1, LGSN, and LIMS2 MAP3K2, MFSD6, and MSTN MYO7B, NAB1, NIPA1, and NIPA2 OCA2, ORMDL1, and OSGEPL1 PLEKHB2, PMS1, and POLR2D PROC, PTPN18, SAP130, SFT2D3, SLC40A1, and TMEM194B TUBGCP5, UGGT1, and WDR33 WDR75

Abbreviations: Chr, chromosome; Start bp, start position in base pairs; End bp, end position in base pairs; nSNPs, number of single-nucleotide polymorphisms.

Key candidate genes identified within this hotspot include myostatin (*MSTN*), a gene associated with double muscling trait of Texel sheep, and collagen type V alpha 2 chain (*COL5A2*), which contributes to tissue structure and development. Genes related to immune function were also identified, such as interferon regulatory factor 6 (*IRF6*) and phosphatidylinositol glycan anchor biosynthesis class P (*PIGP*). Additionally, nuclear pore complex protein 43 (*NUP43*) and tubulin gamma complex-associated protein 5 (*TUBGCP5*) were detected, which are involved in cellular processes and stress response.

### LD blocks and QTL annotation analysis of the ROH hotspot

**FIGURE 5:**
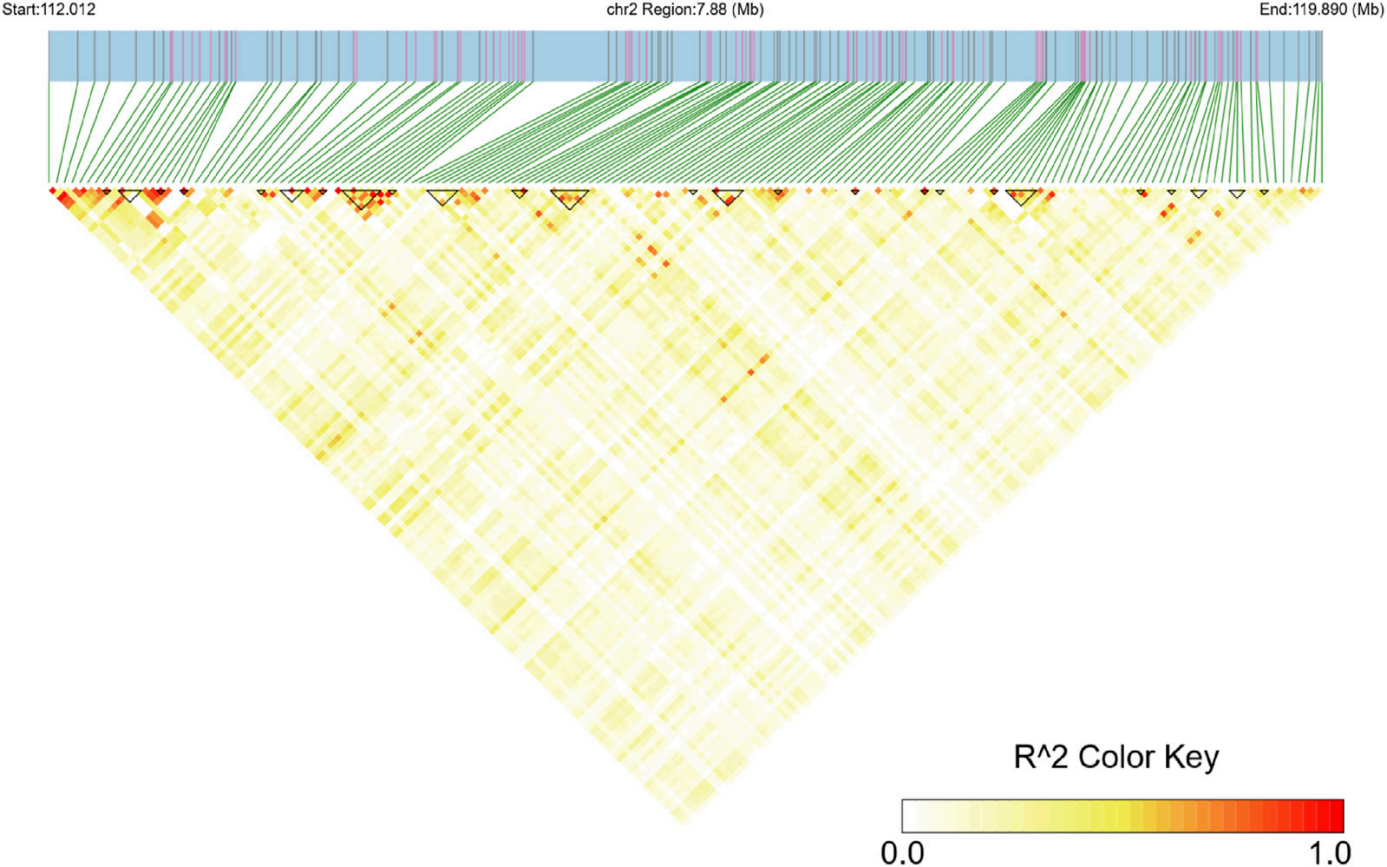
Linkage disequilibrium (LD) heatmap of the ROH hotspot region on chromosome 2. The x-axis and y-axis represent SNP positions in the region, and the color key shows the R2 value, with red indicating high LD and yellow indicating low LD.

To investigate the 7.88 Mb hotspot region on chromosome 2, an LD heatmap was constructed to illustrate linkage disequilibrium patterns within the region. The analysis revealed significant LD block structures, with 24 LD blocks being identified, ranging in size from 9.6 kb to 199.5 kb (Figure 5).

**FIGURE 6:**
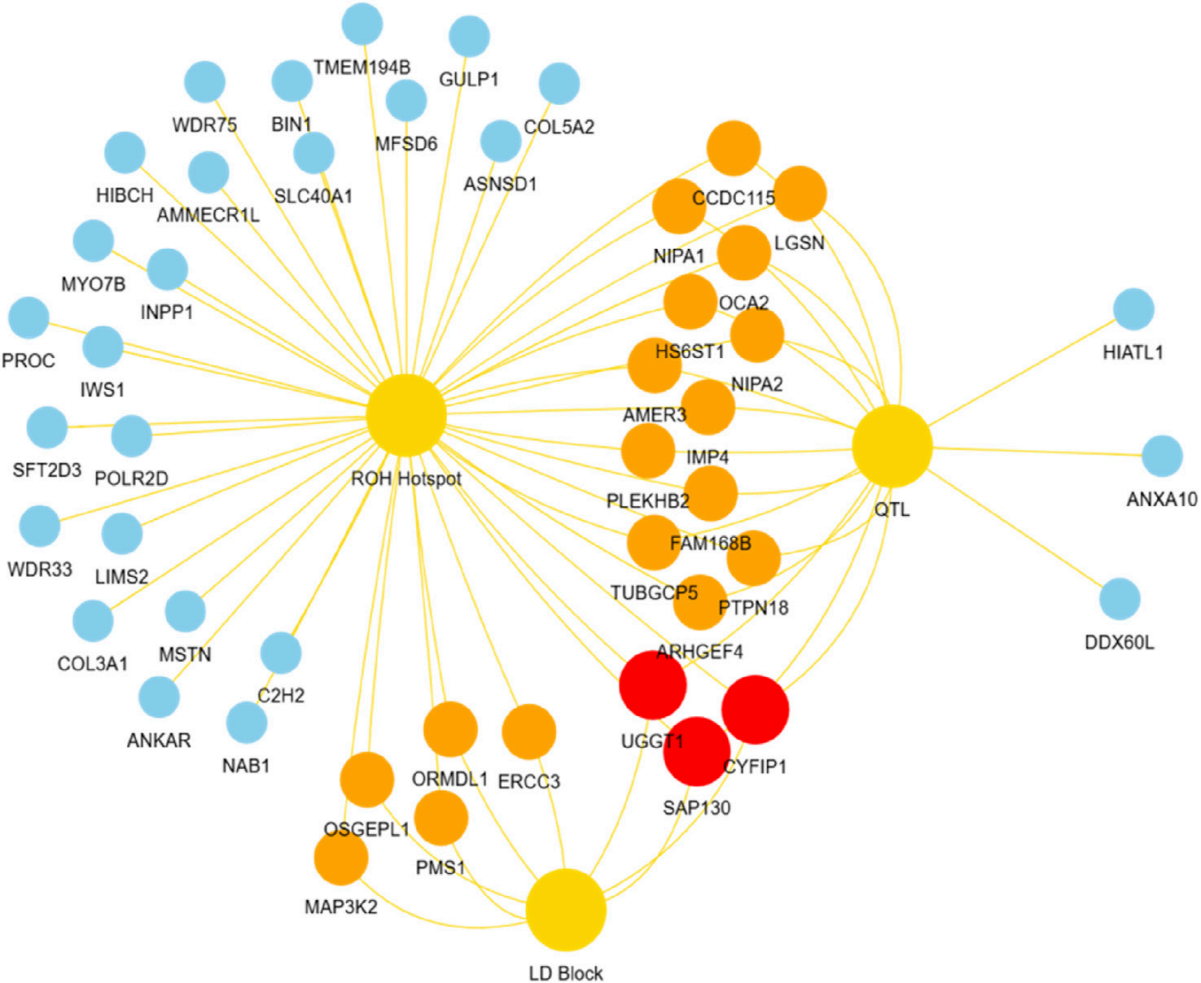
Overlap of candidate genes identified in ROH hotspot, QTL, and LD block. Red nodes represent key candidate genes shared across all three regions. Orange nodes indicate genes shared between any two of the three regions. Blue nodes represent genes unique to each of these regions.

Based on the LD block analysis, QTL annotation analysis was performed to identify regions associated with economically important traits. Two QTLs were identified, both significantly linked to the body condition score (QTL 263631). These QTLs were located at positions 111,201,892–114,207,253 bp and 114,746,188–116,378,689 bp, with lengths of 3.01 Mb and 1.63 Mb, respectively, encompassing 25 and 36 SNPs (Table 5). Further analysis of the candidate genes within the ROH hotspot, QTL, and LD block regions revealed distinct overlaps and unique genes across the three regions. A total of 36 candidate genes were identified. Red nodes represent three key candidate genes shared across all three regions (ROH hotspot, QTL, and LD blocks): Cytoplasmic FMR1 Interacting Protein 1 (*CYFIP1*), Sin3A Associated Protein 130 (*SAP130*), and UDP-Glucose Glycoprotein Glucosyltransferase 1 (*UGGT1*). Orange nodes indicate genes shared between any two regions, including Non-Imprinted in Prader–Willi/Angelman Syndrome 1 (*NIPA1*), Non-Imprinted in Prader–Willi/Angelman Syndrome 2 (*NIPA2*), Heparan Sulfate 6-O-Sulfotransferase 1 (*HS6ST1*), Tubulin Gamma Complex Associated Protein 5 (*TUBGCP5*), and APC Membrane Recruitment Protein 3 (*AMER3*). Blue nodes represent genes unique to a specific genomic region, such as Myostatin (*MSTN*), Ankyrin Repeat Domain 22 (*ANKAR*), Collagen Type V Alpha 2 Chain (*COL5A2*), Engulfment Adapter PTB Domain Containing 1 (*GULP1*), and Bridging Integrator 1 (*BIN1*, Figure 6).

**TABLE 5:** Summary of QTL loci and candidate genes harbored within the ROH hotspot.

Start bp	End bp	Length (Mb)	nSNPs	Associated trait	Genes
111201892	114207253	3.01	25	Body condition score (QTL 263631)	DDX60L, ANXA10, HIATL1, and LGSN OCA2 and NIPA1 NIPA2 and CYFIP1 TUBGCP5 CCDC115 and IMP4 PTPN18, AMER3, and ARHGEF4 FAM168B PLEKHB2
114746188	116378689	1.63	36	Body condition score (QTL 263631)	HS6ST1 and UGGT1 SAP130

## Discussion

Genetic diversity is a critical factor influencing population adaptability and long-term sustainable production, especially for livestock undergoing artificial selection. In this study, multiple genetic diversity parameters characterized the genetic structure of the Teha sheep population. The Ho (0.347), He (0.347), and PIC (0.345) values collectively indicate balanced and robust genetic variability within the Teha sheep population. Notably, the Ho of Teha sheep surpasses that of several indigenous breeds, including the Yongdeng Qishan sheep (Ho = 0.199) and Lanzhou fat-tailed sheep (Ho = 0.341), suggesting superior allelic diversity (Ma KY. et al., 2024). Populations with Ho values ≥0.30 are widely recognized as genetically healthy, capable of maintaining adaptability, and reducing the risks of inbreeding depression and genetic drift (Allendorf et al., 2012; Harmon and Braude, 2010). Maintaining neutral genetic diversity above certain thresholds is essential for long-term population viability in livestock (Harmon and Braude, 2010). In addition, the low FROH (0.044) and relatively large Ne (214.89) further reflect the genetic robustness of the Teha sheep population, which supports maintaining the genetic health and adaptive capacity essential for ongoing genetic improvement (Ma K. et al., 2024). The moderate PIC (0.345) indicates notable genetic variation within the population. A moderate mean MAF (0.253) across the analyzed loci suggests a substantial proportion of medium-frequency alleles. Collectively, these genetic characteristics indicate that the population maintains a favorable level of genetic variability, providing a basis for sustained breeding progress and enhanced production efficiency (Zhu et al., 2024).

The analysis of inbreeding coefficients in the Teha sheep population revealed consistently low levels across both pedigree-based (FPED = 0.001) and genome-based estimates, including FROH (0.044), FHOM (−0.007), FHAT1 (−0.050), FHAT2 (−0.006), and FHAT3 (−0.006). Whereas FPED and FROH are conventional measures of inbreeding, FHOM and FHAT coefficients, derived from genomic data, provide additional insights into the population’s genetic structure. Notably, the negative values observed for FHOM and FHAT1-3 indicate an excess of heterozygosity compared to the expected heterozygosity under the Hardy–Weinberg equilibrium, which is potentially due to recent population admixture or selection pressures favoring genetic diversity (Alemu et al., 2021; Caballero et al., 2022). Studies confirm that negative inbreeding coefficients can reflect higher than expected heterozygosity under such conditions, often resulting from population mixing or selection (Lukić et al., 2020). The low FROH value (0.044) in Teha sheep is lower than that of crossbred Black Slavonian pigs (FROH = 0.098) and comparable to locally adapted breeds such as Simmental cattle (FROH = 0.059) (Visser et al., 2023), highlighting their genetic diversity. These findings underscore effective breeding management practices that maintain low inbreeding levels and high genetic diversity, which is crucial for Teha sheep’s long-term viability and productivity. Thus, these results provide a foundation for developing sustainable breeding strategies that preserve genetic health.

ROH hotspots are genomic regions marked by high homozygosity and are frequently linked to selection pressures and traits associated with adaptability and fitness (Lee et al., 2013). These regions have been widely identified in livestock species such as cattle and sheep, underscoring their pivotal role in adaptive evolution and economically important production traits. Our work identified a primary ROH hotspot in Teha sheep on chromosome 2, spanning 112,011,937–119,890,211 base pair, with a length of 7.88 Mb and encompassing 184 SNPs. This notable clustering of SNPs underscores the potential role of harbored genes associated with growth and adaptability, offering crucial insights into the genetic framework of Teha sheep.

The ROH hotspot on chromosome 2 contains key genes linked to muscle growth, fat deposition, and skeletal development, all of which are essential for production efficiency and adaptability. *MSTN* stands out as a well-documented regulator of muscle growth, with its mutations associated with the double muscling phenotype in Texel sheep and Belgian Blue cattle, leading to significantly enhanced meat yields (Garcia et al., 2019; Aiello et al., 2018). Similarly, *COL5A2* plays a critical role in connective tissue integrity, contributing to carcass strength and quality, whereas C2H2ORF88 is involved in muscle fiber organization and regeneration, supporting skeletal robustness (Harris et al., 2023; Ramljak et al., 2024). ROH analysis, as an effective tool, has been widely applied in sheep genomics studies to identify selection signatures underlying specific traits. For instance, a prominent ROH island on *Ovis aries* chromosome 6 (OAR6) was identified in French dairy sheep through ROH analysis, and it was associated with milk production traits (Rodríguez-Ramilo et al., 2021). Such studies indicate that different sheep breeds may experience specific selection pressures on distinct chromosomal regions due to differing breeding objectives. The ROH hotspot identified on chromosome 2 in Teha sheep in our study suggests potential selection targeting its meat traits. These findings further support a strong genetic foundation for production traits in the Teha sheep population and provide a basis for developing sustainable breeding strategies that preserve genetic health.

Fat metabolism-related genes, such as *TUBGCP5* and *PIGP*, were also identified within the hotspot. *TUBGCP5* regulates the intramuscular fat content, which is critical for meat quality in Hanwoo cattle, whereas *PIGP* influences the fat distribution in Merino sheep (Garcia et al., 2019; Aiello et al., 2018). Additionally, skeletal development genes, including *NIPA1* and *NIPA2*, were enriched in this region, underscoring their role in bone mineralization and structural strength (Harris et al., 2023). The functional significance of these genes highlights the selective pressures acting on this region and their importance in Teha sheep’s adaptive and productive traits.

In this study, we further integrated QTL annotation analysis and LD block analysis, revealing two significant QTLs (QTL 263631) strongly associated with the ROH hotspot on chromosome 2. Both QTLs were linked to the body condition score, located at positions 111,201,892–114,207,253 bp and 114,746,188–116,378,689 bp, spanning 3.01 Mb and 1.63 Mb, and containing 25 and 36 SNPs, respectively. The LD block analysis demonstrated substantial overlap with these QTL regions, with LD blocks ranging in size from 9.6 kb to 199.5 kb (Figure 5). This significant overlap underscores the evolutionary importance of these loci under strong selective pressure, particularly for traits related to growth and production (Ramljak et al., 2024).

Through comprehensive candidate gene analysis, *CYFIP1*, *SAP130*, and *UGGT1* were identified as key genes shared across the ROH hotspot, QTLs, and LD blocks, directly implicating their functions in growth, carcass quality, and fat deposition. For instance, *CYFIP1* has been shown to regulate growth efficiency and carcass weight, with evidence from studies in Charolais cattle (Colombi et al., 2024). *SAP130* plays a crucial role in adipose metabolism and muscle development, exhibiting strong associations with carcass quality in various cattle breeds (Colombi et al., 2024). *UGGT1*, on the other hand, is essential for protein quality control under cellular stress and has been linked to meat quality traits in Hanwoo cattle and other livestock species (Mastrangelo et al., 2018).

Although we provide important insights into the genetic diversity and selection signals of the Teha sheep population in this study, certain limitations should be noted. The analysis was based on a single population with a limited sample size, which may not fully represent the genetic structure of the broader population. Previous studies have demonstrated that the population structure and sampling strategy can significantly influence estimates of ROH and genetic variability (Peripolli et al., 2017; Ceballos et al., 2018). Additionally, during the analysis, it is noteworthy that ROH information for chromosomes 24, 26, and the end of chromosome 23 could not be presented in Figures 2C, 3C, 4. This absence does not imply a biological lack of ROHs in these regions but rather stems from various technical limitations encountered during the original SNP chip data processing, such as insufficient local SNP density or high genotype missingness, which precluded reliable ROH detection and visualization for these specific areas. Nevertheless, the core findings of this study, particularly the identification of major ROH hotspots, are based on chromosomal regions with complete and highly reliable SNP data. Consequently, these localized data limitations do not substantially affect the validity and generality of our main conclusions. Future studies incorporating multiple populations and higher-resolution data such as whole-genome sequencing would help validate and extend our findings.

Moreover, the ROH hotspots and candidate genes identified here offer promising targets for genomic selection. Incorporating these regions into marker-assisted or genomic selection frameworks could accelerate genetic gains in traits related to growth, carcass quality, and adaptability. Several studies have already shown the practical use of ROH-based selection in livestock breeding programs, supporting the potential of this approach in Teha sheep and other crossbred populations (Peripolli et al., 2017).

## Conclusion

In this study, we provide a comprehensive evaluation of genetic diversity, inbreeding patterns, and selection signals in Teha sheep, a breed adapted to the unique environmental conditions of Xinjiang, China. The results reveal low levels of inbreeding through pedigree and genomic data, highlighting the population’s high genetic variability. A significant ROH hotspot was identified on chromosome 2, harboring key genes associated with economically important traits, such as muscle growth, fat metabolism, and skeletal development. These findings highlight the utility of genomic approaches in dissecting functional variation underlying complex traits. The identified ROH regions and associated candidate genes offer actionable targets for genomic selection and conservation efforts in Teha sheep and related crossbred populations. We advocate for the integration of such genomic insights into the design of data-driven breeding strategies aimed at enhancing long-term productivity and environmental resilience in livestock systems.

## Data Availability

The data analyzed in this study is subject to the following licenses/restrictions: datasets belong to Xinjiang Academy of Animal Science. Requests to access these datasets should be directed to liumingjun@xjaas.net.

## Abbreviations

FPED, pedigree-based inbreeding coefficient; FROH, runs of homozygosity-based inbreeding coefficient; Ho, observed heterozygosity; He, expected heterozygosity; PIC, polymorphic information content; ROH, runs of homozygosity; QTL, quantitative trait loci; LD, linkage disequilibrium; MAF, minor allele frequency; Ne, effective population size; ENA, effective number of alleles; FHAT1, inbreeding coefficient based on excess variance standardization; FHAT2, inbreeding coefficient based on excess homozygosity; FHAT3, inbreeding coefficient based on allele correlation within gametes; FROHOAR, chromosome-specific inbreeding coefficients; FHOM, inbreeding coefficient based on the proportion of homozygous genotypes; SNPs, single-nucleotide polymorphisms.
