# Supplementary figures and images for: Inbreeding patterns and genetic diversity under selection in Teha sheep
Source: Front Genet. 2025 Jun 27;16:1576125. doi: 10.3389/fgene.2025.1576125 (PMC12245786; doi:10.3389/fgene.2025.1576125)

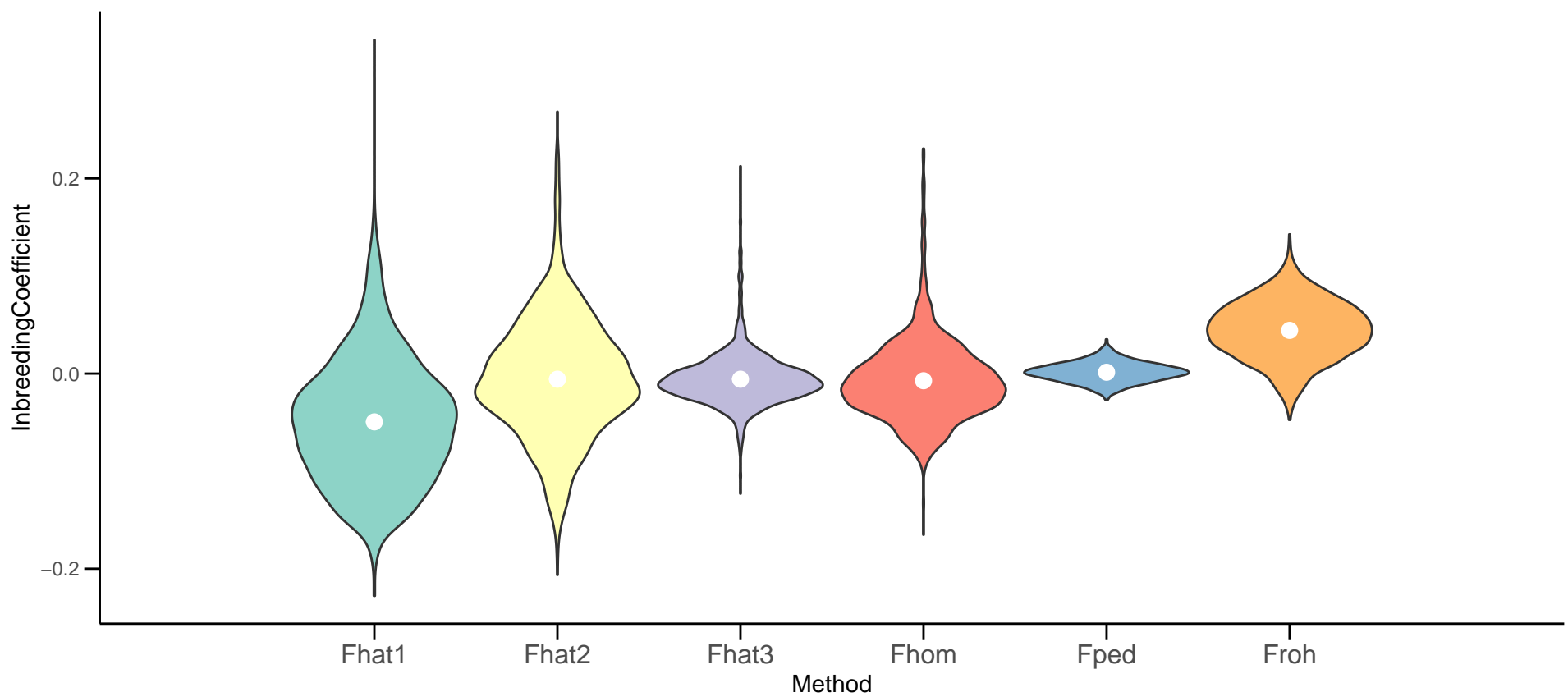

Supplement: Supplementary file 1 [file DataSheet1.zip › Raw data/HOM&HAT/Violin diagram.pdf]

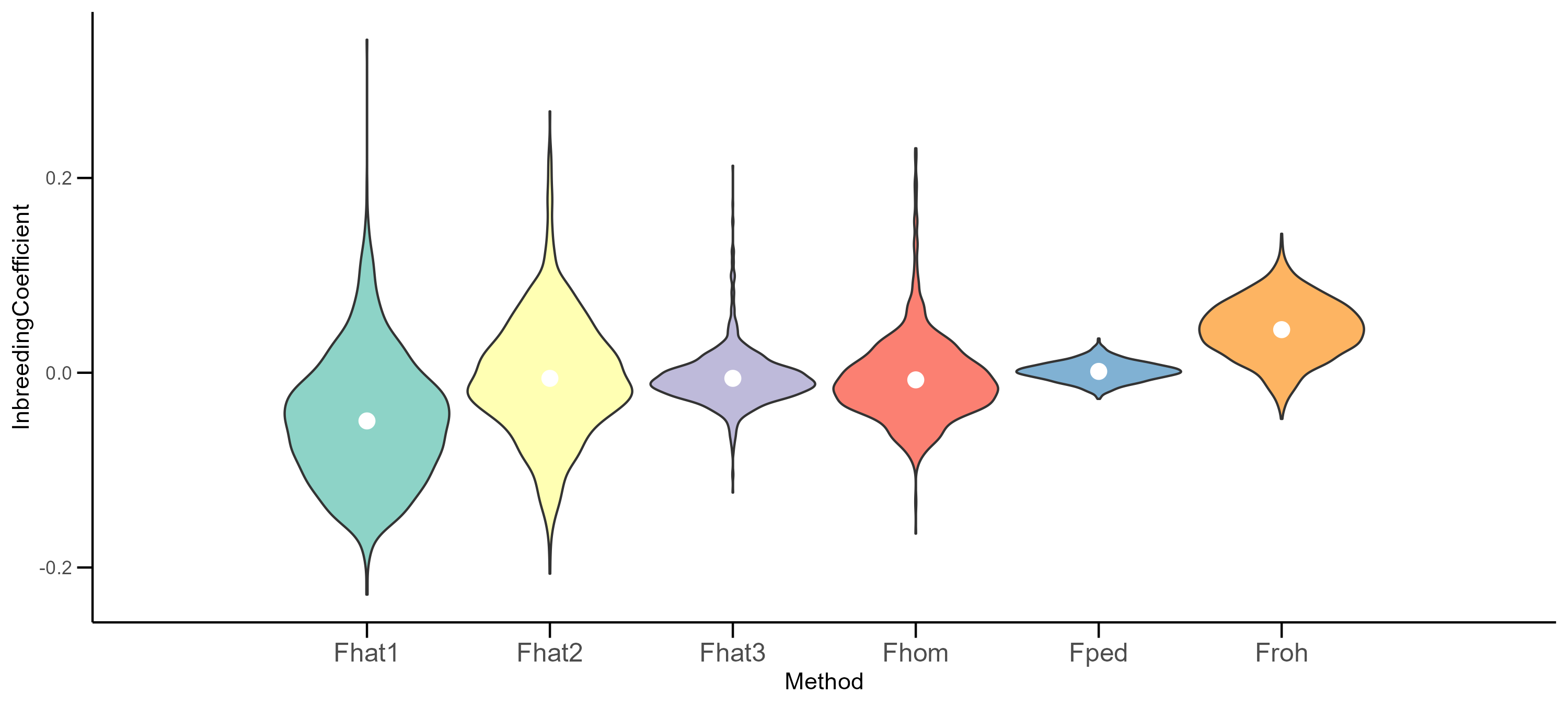

Supplement: Supplementary file 1 [file DataSheet1.zip › Raw data/HOM&HAT/Violin diagram.png]
