# Supplementary material for: Inbreeding patterns and genetic diversity under selection in Teha sheep
Source: Front Genet. 2025 Jun 27;16:1576125. doi: 10.3389/fgene.2025.1576125 (PMC12245786; doi:10.3389/fgene.2025.1576125)
Supplement: Supplementary file 2 [file DataSheet2.docx]

**SNP Data Quality Control**

A total of 1,273 samples and 64,734 SNPs were initially included in the analysis. After quality control (QC), 1,271 samples and 56,297 high-quality SNPs were retained, with 2 samples and 8,437 SNPs removed. The sample missing rates (F_MISS) before quality control were mostly concentrated below 0.03, with a few outliers reaching up to approximately 0.06. After QC, the missing rates were significantly reduced, with nearly all samples showing missing rates close to zero and only a very small number of outliers exceeding 0.01 (S1). These changes highlight the effectiveness of QC in improving the reliability of SNP data for subsequent analyses.


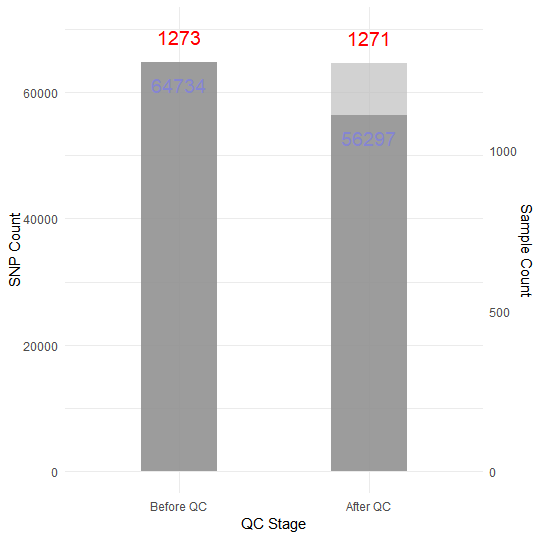

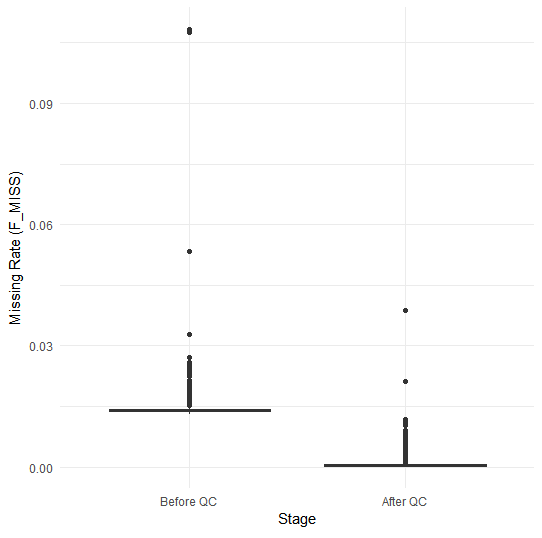


**A**

**B**

**S1.** SNP and sample quality control (QC). (A) SNP and sample counts before and after QC. The red numbers indicate the total number of samples before and after QC, while the blue numbers represent the total number of SNPs retained before and after QC. (B) Sample missing rates (F_MISS) before and after QC.
